# Vitamin D supplementation lowers thrombospondin-1 levels and blood pressure in healthy adults

**DOI:** 10.1371/journal.pone.0174435

**Published:** 2017-05-10

**Authors:** Anjalee T. Amarasekera, Bahador Assadi-Khansari, Saifei Liu, Marilyn Black, Greer Dymmott, Natasha M. Rogers, Aaron L. Sverdlov, John D. Horowitz, Doan T. M. Ngo

**Affiliations:** 1 Cardiology Unit, Basil Hetzel Institute, The Queen Elizabeth Hospital, Department of Medicine, The University of Adelaide, Adelaide, South Australia, Australia; 2 School of Pharmacy and Medical Sciences, University of South Australia, Adelaide, South Australia, Australia; 3 Centre for Transplant and Renal Research, Westmead Institute for Medical Research, Sydney, New South Wales, Australia; Center for Cancer Research, UNITED STATES

## Abstract

**Introduction:**

Vitamin D insufficiency, defined as 25-hydroxyvitamin D (25(OH)D) levels < 75nmol/L is associated with cardio-metabolic dysfunction. Vitamin D insufficiency is associated with inflammation and fibrosis, but it remains uncertain whether these anomalies are readily reversible. Therefore, we aimed to determine the effects of vitamin D supplementation on markers of: 1) nitric oxide (NO) signaling, 2) inflammation, and 3) fibrosis, in healthy volunteers with mild hypovitaminosis.

**Methods:**

Healthy volunteers (n = 35) (mean age: 45 ± 11 years) with 25(OH)D levels <75nmol/L, received vitamin D supplementation (Ostelin ® capsules 2000IU) for 12 weeks. Resting systolic and diastolic blood pressures (BP) were assessed. Routine biochemistry was examined. Plasma concentrations of asymmetric dimethylarginine (ADMA), thrombospondin-1 (TSP-1), plasminogen activator inhibitor-1 (PAI-1), hs-CRP, activin-A, and follistatin-like 3 (FSTL3) were quantitated.

**Results:**

Vitamin D administration for 12 weeks significantly increased 25-(OH)D levels (48.8 ± 16 nmol/L to 100.8 ± 23.7 nmol/L, p<0.001). There was significant lowering of systolic and diastolic BP, while there was no significant change in lipid profiles, or fasting insulin. Plasma concentrations of ADMA, hs-CRP, PAI-1, activin A, and FSTL-3 did not change with vitamin D supplementation. However, there was a marked reduction of TSP-1 (522.7 ± 379.8 ng/mL vs 206.7 ± 204.5 ng/mL, p<0.001).

**Conclusions:**

Vitamin D supplementation in vitamin D insufficient, but otherwise healthy individuals markedly decreased TSP-1 levels and blood pressure. Since TSP-1 suppresses signaling of NO, it is possible that the fall in BP is engendered by restoration of NO effect.

## Introduction

Vitamin D deficiency (25-hydroxyvitamin D (25(OH)D) < 50nmol/L) or insufficiency (25(OH)D <75nmol/L, is present in approximately 30% to 50% of the general population, and is suggested to contribute to the pathogenesis of numerous diseases [1]. Similarly, the Third National Health and Nutrition Examination Survey (NHANES) data suggested the occurrence of vitamin D deficiency in the United States to be 25% to 57% of adults [2].

It is increasingly reported that vitamin D status is important in cardiovascular homeostasis [3–6], and conversely, that hypovitaminosis D may represent an independent risk factor for the development of coronary disease [7, 8]. To date, the mechanism(s) underlying the putative detrimental effects of hypovitaminosis D on cardiovascular function is unclear. Hypovitaminosis D is associated with inflammatory activation[9], endothelial[10] and platelet dysfunction[11], as well as fibrosis[12]. While there are numerous studies of an association between low vitamin D status and various disease states; there are only limited numbers of studies on the effects of vitamin D supplementation, particularly in young otherwise healthy adults. In particular, the effects of vitamin D supplementation on biomarkers of vascular function and thrombogenicity are limited. Previously, we have found an inverse relationship between low 25(OH)D levels and plasma concentrations of asymmetric dimethylarginine (ADMA), a marker of impaired nitric oxide (NO) synthase activity [10]. The effects of vitamin D supplementation on ADMA concentrations are unknown. In the current study of vitamin D insufficient, but otherwise healthy volunteers, we determined the effects of vitamin D supplementation on biomarkers of NO signaling: 1) ADMA, 2) thrombospondin-1 (TSP-1), and 3) platelet NO responsiveness. Given the postulated role of vitamin D in inflammatory activation, fibrosis and hemostasis, we also examined whether normalization of vitamin D would affect related biological markers such as hs-CRP, activin A, follistatin-like 3 (FSTL3), and plasminogen-activator inhibitor-1 (PAI-1).

## Methods

### Study subjects

Healthy volunteers were consecutively recruited by advertisement from within The Queen Elizabeth Hospital, Woodville, South Australia. Clinical history of each subject was evaluated by qualified medical practitioner before enrolment to the study. None of the subjects were receiving vitamin D supplementation at the time of recruitment. The study complied with the Declaration of Helsinki and was approved by the Ethics of Human Research Committee of The Queen Elizabeth Hospital. All study subjects provided informed written consent on the patient information document approved by the Ethics of Human Research Committee of The Queen Elizabeth Hospital.

**Fig 1:**
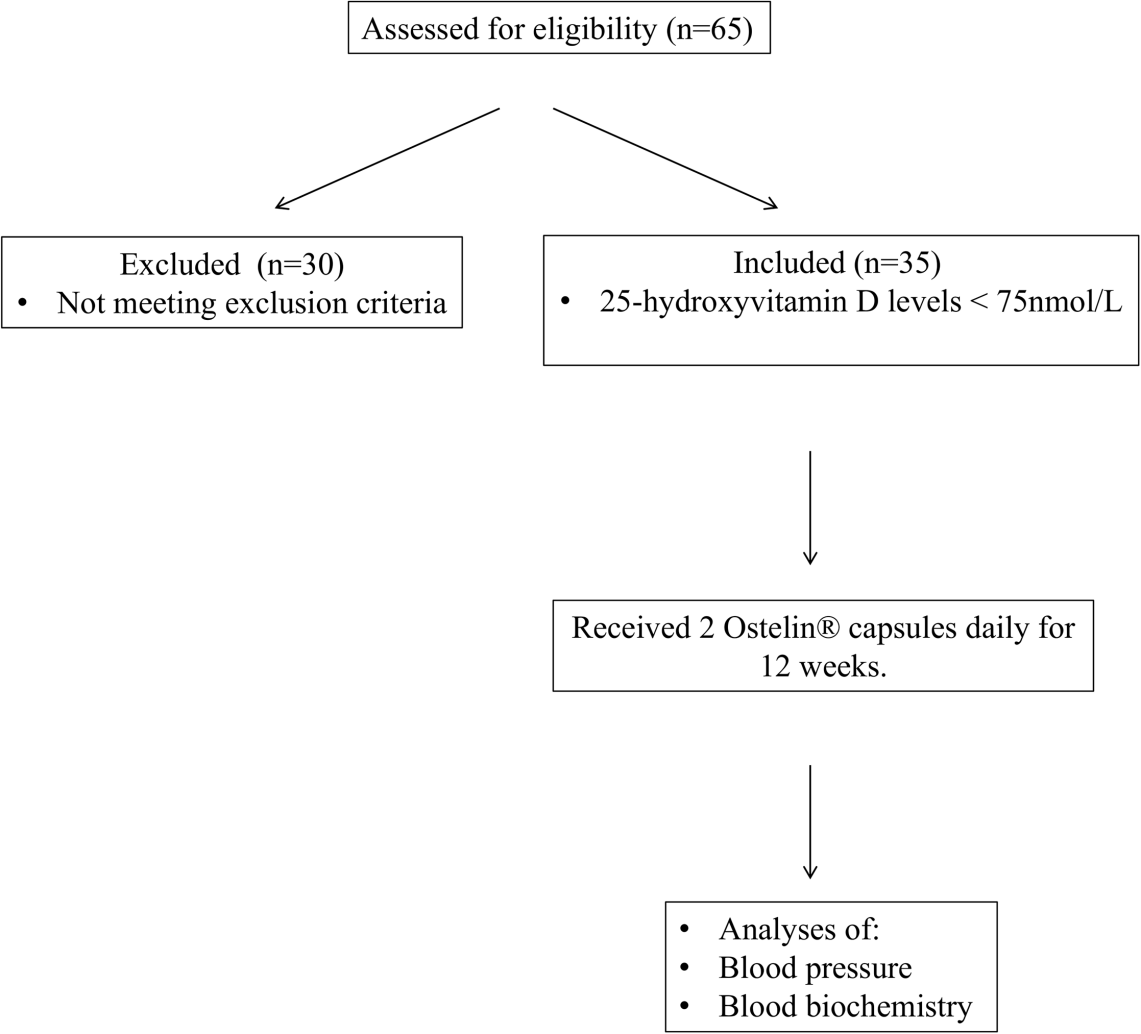
Study design diagram.

In response to an invitation to participate, sixty-five (n = 65) healthy volunteers without any pre-existing heart disease, diabetes mellitus or liver / renal insufficiency were screened for vitamin D insufficiency. Of those, thirty-five (mean age: 45 ± 11 years) were diagnosed with vitamin D insufficiency, defined as plasma 25-hydroxyvitamin D (25(OH)D) levels < 75 nmol/L [1]. This was the sole criterion for selection. Fig 1 depicts the study design. Participants were enrolled consecutively within the same season, Spring; with subsequent follow up periods in Summer.

Height, weight, and body mass index (BMI) were recorded for all these subjects at study entry and at a follow up visit. Fasting blood samples were collected for biochemical parameters as described below. Vitamin D_3_ capsules (Ostelin® vitamin D capsules (Australia)) were dispensed from the hospital pharmacy at a dose of 2000 IU of vitamin D_3_ (two capsules of 1000 IU) daily for 12 weeks. At the end of the 12 weeks of treatment, the subjects were re-evaluated for the same parameters as at baseline. Compliance was assessed by the percentage of prescribed pills ingested. All patients had at least 80% compliance rate.

### Biochemical and physiological measures

Fasting blood was taken via an antecubital vein for the determination of biochemical parameters including fasting lipid profile, high-sensitivity C-reactive protein (hs-CRP), 25(OH)D levels, glucose and insulin levels. The quantitative homeostatic model assessment (HOMA) was used as a surrogate index of insulin resistance. 25(OH)D levels were assayed by commercially available radioimmunoassay after extraction (Immunodiagnostic Systems, Boldon,UK) as in our previous studies [10, 13]. Peripheral blood was collected into sodium heparin tubes and placed immediately on ice. Non-hemolysed, platelet-poor plasma was generated by centrifugation at 1,800 g for 15 min at 4°C, with the supernatant centrifuged at 10,000 g for 10 min at 4°C, then stored at −70°C until analysis. Plasma concentrations of thrombospondin-1 (TSP-1) were determined by solid-phase ELISA (enzyme-linked immunosorbent assay) (Quantikine ELISA human thrombospondin-1 immunoassay kit, R & D systems, Minneapolis, MN, USA). Plasma concentrations of other biomarkers were determined using commercially available ELISA kits: PAI-1 (ThermoFisher), Activin-A (R&D systems), and Follistatin-like 3 (R&D systems) were also quantitated. Plasma concentrations of asymmetric dimethylarginine (ADMA), a marker and mediator of endothelial dysfunction [14], were determined by high-performance liquid chromatography with the derivatization reagent AccQ-Fluor (Waters, Milford, MA, USA) after solid phase extraction, as previously described [10].

### Platelet aggregometry

Platelet aggregometry was performed using whole-blood impedance aggregometry, as previously described [15]. Briefly, venous blood was collected from an antecubital vein into 10-ml tubes containing 1:10 volume of acid citrate anticoagulant (2 parts 0.1 M citric acid to 3 parts of 0.1 M trisodium citrate). Aggregation was induced with ADP (2.5 μM), and responses were recorded for electrical impedance (Ω) via a computer interface system (Aggrolink, Chrono-Log, Havertown, PA), as previously described [15].

### Blood pressure measurements

Resting blood pressure was measured after subjects are rested for a minimum of 30 minutes. All measurements were performed three times by keeping five minute time gap between the measurements and the average of the three measurements was utilized for analysis at each visit. Blood pressure was performed unblinded, by the same investigator before and after vitamin D supplementation using the same electronic machine (Omron Digital Automatic Upper Arm Blood pressure monitor).

### Statistical analyses

The primary endpoint was change in ADMA concentrations from baseline to 12 weeks of vitamin supplementation. Our preliminary mean plasma ADMA concentrations in vitamin D deficient volunteers were: 0.55 ± 0.1μM [10]. Therefore, 33 subjects will be required to have 80% power to detect 0.5SD difference induced by treatment that is a change of 0.05μM.

All data are expressed as mean ± SD unless otherwise stated. All continuous variables were tested for Gaussian distribution, and skewed data were normalized by either log or square root transformation prior to linear regression analyses. Baseline and end-of-study parameters were compared by paired t-test (for normally distributed data) or Wilcoxon matched-pair signed-rank test (for non-parametric data). All t-tests were two-sided. Linear regression analyses were performed to assess the relationships between change in 25(OH)D levels and biochemical/physiological parameters. All analyses were performed with the SPSS version 20 software (SPSS, Chicago, IL, USA), and a *P*-value of < 0.05 was considered to be statistically significant.

## Results

### Baseline subject characteristics before and after vitamin D supplementation

**Fig 2:**
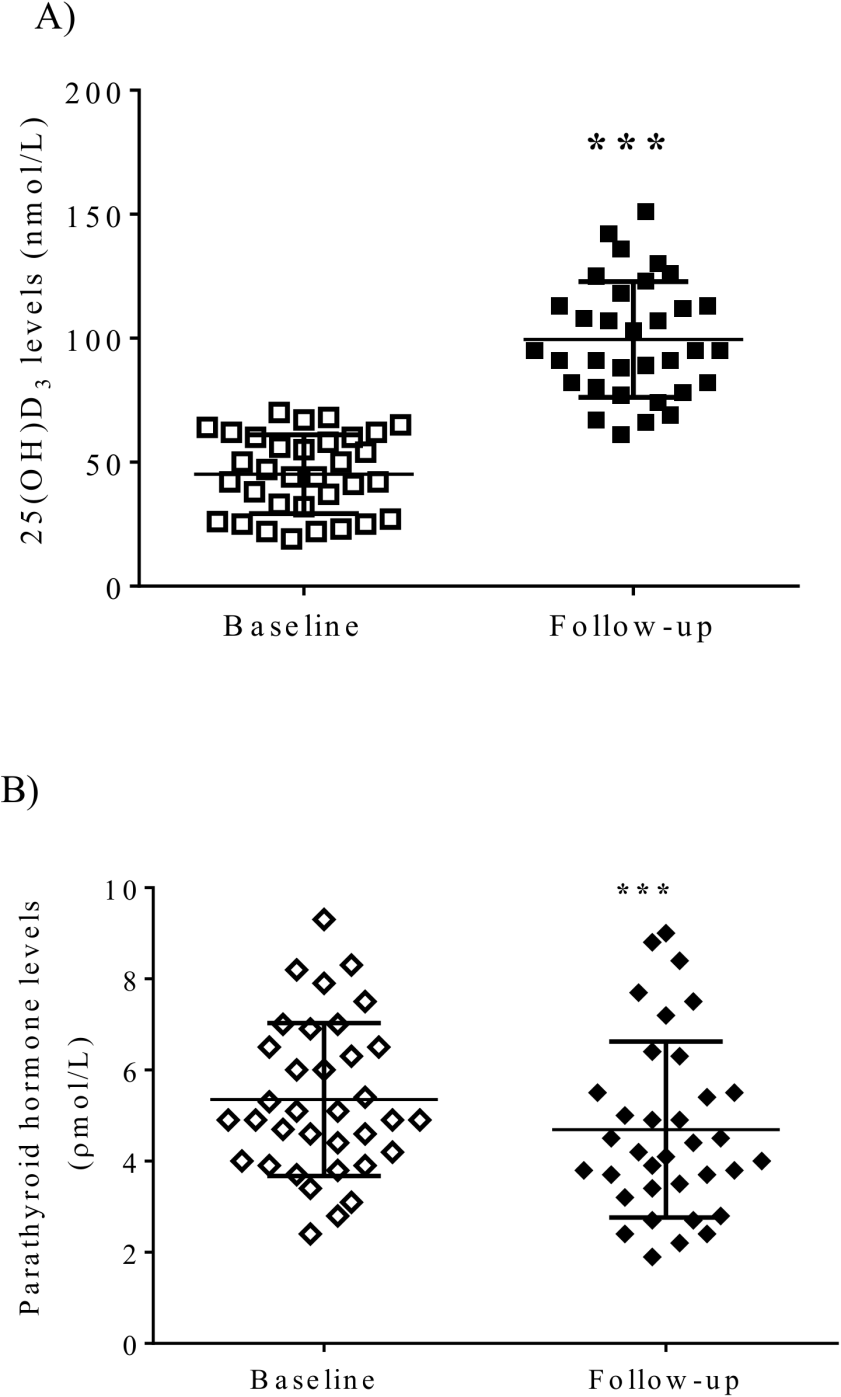
Significant changes of A) vitamin D levels and B) PTH levels with vitamin D replacement therapy in healthy volunteers. ***p<0.001.

**Fig 3:**
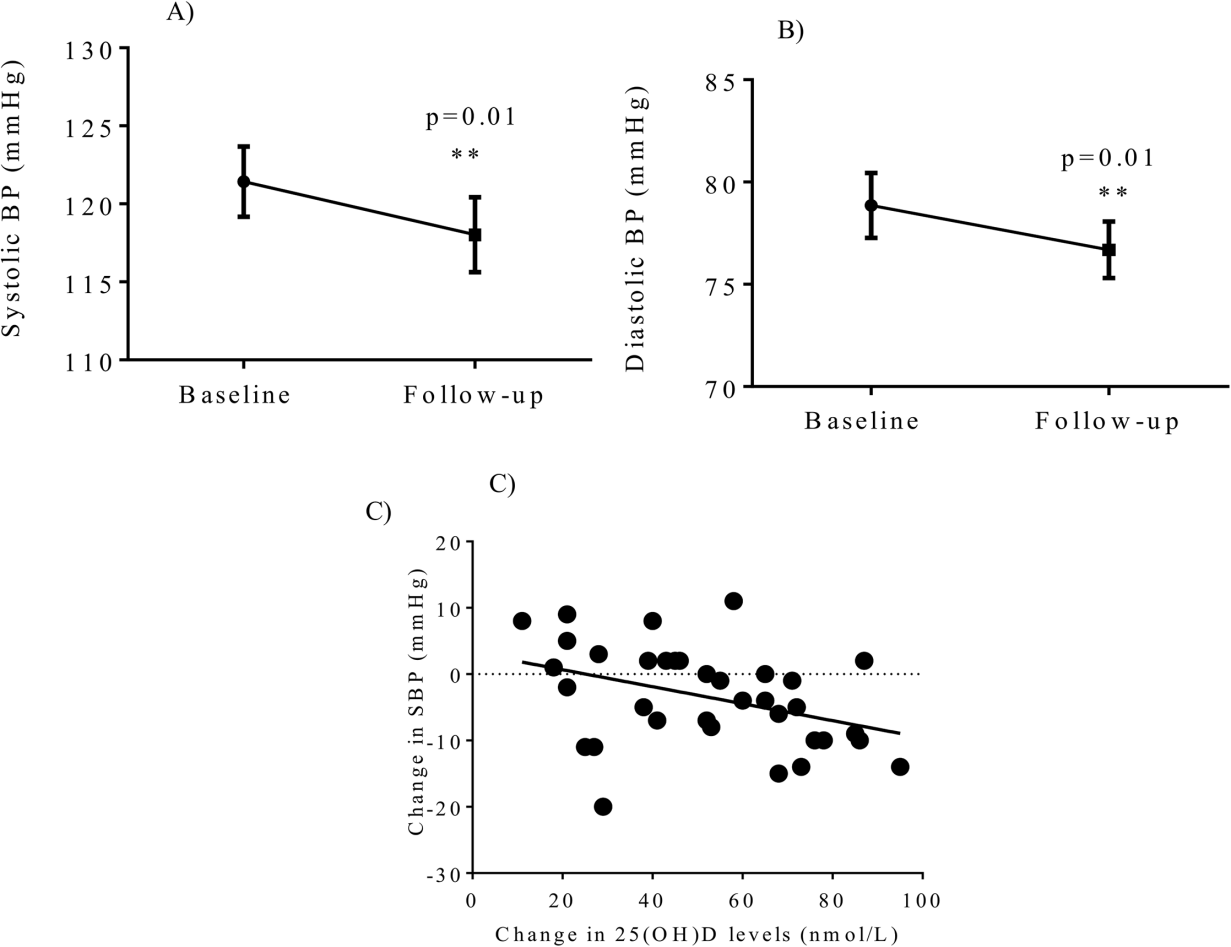
Vitamin D supplementation after 12 weeks significantly reduced (A) systolic blood pressure, and (B) diastolic blood pressure. There was a significant correlation between an increase in 25-hydroxyvitamin D levels and lowering of systolic blood pressure (C).

Study parameters at baseline and follow up visits of these vitamin D insufficient healthy subjects are shown in **Table 1**. Vitamin D administration for 12 weeks significantly increased 25(OH)D levels compared with baseline values (baseline 48.8 ± 16 nmol/L vs follow up 100.8 ± 23.7 nmol/L, p<0.001) **(Fig 2A)** and reduced PTH levels (baseline 5.4 ± 1.7 pmol/L, vs follow-up 4.7 ± 1.9 pmol/L, follow-up, p<0.001) **(Fig 2B)**. At the follow up visits 31/35 (86%) of subjects increased their serum 25(OH)D levels to the normal range (> 75 nM). There was no significant difference in total calcium, ionised calcium, phosphate levels and calcium-phosphate product, but borderline reduction of fasting glucose levels (p = 0.05) was seen. However, fasting insulin levels (p = 0.1) or insulin sensitivity measured by HOMA (p = 0.1) were not significantly improved after the vitamin D administration. Vitamin D administration significantly reduced systolic blood pressure (p = 0.007) **(Fig 3A)** and diastolic blood pressure (p = 0.008) levels **(Fig 3B)**. None of the other parameters tested were significantly improved after the vitamin D treatment including lipid profiles and markers of inflammation **(Table 1)**

**Table 1:** Clinical characteristics of subjects at baseline and after 12 weeks of vitamin D supplementation.

	Baseline	Follow-up	p value
25(OH)D_3_ levels (nmol/L)	43.7 ± 16.2	98.5 ± 23.6	<0.001
PTH levels (pmol/L)	5.4 ± 1.7	4.7 ± 1.9	<0.001
Fasting glucose (mmol/L)	5.2 ± 0.6	5.1 ± 0.6	0.05
Fasting insulin (mU/L)	12.9 ± 7.2	12.0 ± 6.9	0.2
HOMA-IR	3.0 ± 1.7	3.5 ± 4.9	0.3
BMI (kg/m^2^)	28.1 ± 5.6	27.6 ± 7.5	0.5
Ionised calcium (mmol/L)	1.2 ± 0.03	1.2 ± 0.03	0.4
Calcium (mmol/L)	2.4 ± 0.08	2.4 ± 0.08	0.6
Phosphate (mmol/L)	1.11 ± 0.2	1.08 ± 0.1	0.2
Systolic blood pressure (SBP) (mmHg)	121.4 ± 13.3	118.0 ± 14.2	0.01
Diastolic blood pressure (DBP) (mmHg)	78.9 ± 9.4	76.7 ± 8.2	0.01
Total cholesterol (mmol/L)	5.4 ± 1.0	5.3 ± 0.9	0.3
LDL cholesterol (mmol/L)	3.3 ± 0.8	3.2 ± 1	0.7
HDL cholesterol (mmol/L)	1.53 ± 0.4	1.47 ± 0.4	0.09
Triglycerides (mmol/L)	1.6 ± 1.0	1.7 ± 1.1	0.1

### Thrombospondin-1 levels decrease significantly after the treatment with vitamin D

**Fig 4:**
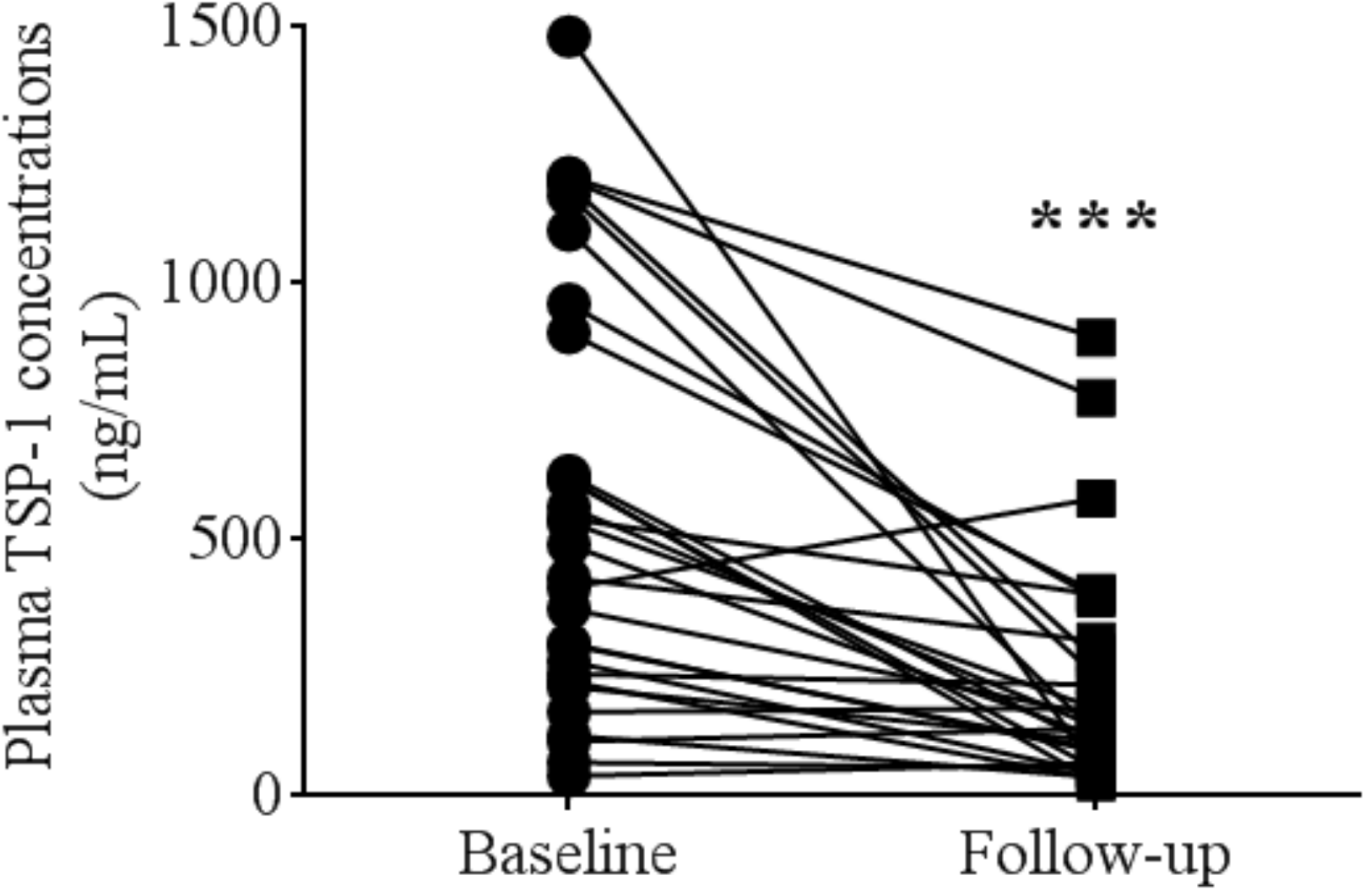
There was a significant reduction of thrombospondin-1 levels after 12 weeks of vitamin D supplementation.

Vitamin D supplementation for 12 weeks markedly reduced TSP-1 levels by almost 2.5 fold (522.7 ± 379.8 ng/mL vs 206.7 ± 204.5 ng/mL, p<0.001) **(Fig 4)**. However, there was no significant change in plasma ADMA concentrations, platelet responsiveness to SNP, PAI-1, Activin-A, or FSTL-3 levels after vitamin D supplementation **(Table 2)**.

**Table 2:** Effects of vitamin D supplementation on changes of nitric oxide, inflammation, and fibrotic biomarkers.

	Baseline	Follow-up	p-value
TSP-1 (ng/mL) **	535.0 (39.0, 2734.0)	134.5 (26.7, 894.2)	<0.001
ADMA (μmol/L)	0.56 ± 0.09	0.57 ± 0.08	0.6
L-arginine (μmol/L)	94.8 ±18.1	97.1 ±18.5	0.4
hs-CRP (mg/L) **	1.8 (0.33, 27)	1.8 (0.32, 19)	0.9
PAI-1 (ng/mL)	1192.7±797	1198.3±855	0.9
Activin-A (pg/mL)	210.3±72	211.6±69	0.9
FSTL-3 (pg/mL)	5927.9±1428	5891.9±1194.2	0.8
Inhibition of platelet aggregation by SNP (%)	18.6 (0, 85)	15.4 (0, 87)	0.1
ADP-induced platelet aggregation (Ω)	8.2±1.9	8.5±2.2	0.5

ADMA = asymmetric dimethylarginine; hs-CRP = hs-C-reactive protein; TSP-1 = thrombospondin-1; SNP = sodium nitroprusside

** Non-normally distributed data are expressed as median (min, max).

### Correlates of change in 25(OH)D levels

**Table 3**depicts correlates of changes in 25(OH)D levels before and after 12 weeks of vitamin D supplementation. The rise in 25(OH)D levels was significantly correlated with lowering of LDL and HDL levels, with a borderline relationship with lowering of total cholesterol. Furthermore, increases in 25(OH)D were also associated with lowering of fasting insulin levels, and systolic blood pressure (**Fig 3C**).

**Table 3:** Linear regression analyses with change in 25-hydroxyvitamin D levels.

Parameter	R- value	p-value
Change in PTH	0.3	0.07
Change in ionised calcium	0.02	0.9
Change in phosphate	0.2	0.3
Change in TSP-1	0.05	0.8
Change in total cholesterol	-0.3	0.05
Change in LDL*	-0.4	0.02
Change in HDL**	-0.5	0.002
Change in triglycerides	0.2	0.3
Change in fasting glucose	-0.03	0.9
Change in insulin*	-0.4	0.04
Change in ADMA	0.3	0.07
Change in Arginine	0.3	0.1
Change in hs-CRP	-0.04	0.9
Change in % inhibition of platelet SNP responsiveness	-0.2	0.2
Change in ADP-induced platelet aggregation	0.05	0.8
Change in PAI-1	0.2	0.3
Change in Activin-A	0.03	0.8
Change in FSTL-3	0.3	0.07
Change in systolic BP*	-0.4	0.02
Change in diastolic BP	-0.3	0.1

*p<0.05

**p<0.01.

## Discussion

This study establishes that adequate vitamin D supplementation (i) markedly decreases TSP-1 concentrations, while it has no effect on ADMA concentrations; ii) lowers blood pressure with a significant correlation between the increase in 25(OH)D levels with supplementation and fall in blood pressure.

A large number of epidemiological studies have suggested that vitamin D status is important in cardiovascular homeostasis [5]. Vitamin D deficiency may represent a risk factor for the development of coronary disease [16], and be an independent risk factor for cardiovascular events in general, independent of conventional cardiovascular risk factors [17], as well as mortality [18]. However, the mechanism underlying the role of vitamin D in cardiovascular outcomes remains largely elusive. Increased oxidative stress and impaired vascular endothelial function have been suggested to be partly responsible for the adverse cardiovascular outcomes associated with low vitamin D in both animal and human studies [19]. Vitamin D has been shown to increase activity and expression of endothelial nitric oxide synthase (eNOS), the enzyme critical to the generation and bioavailability of nitric oxide [20, 21]. In spontaneously hypertensive rats, chronic treatment with active vitamin D metabolite reduced endothelium-dependent vascular contractions in the aorta, decreased blood pressure, and limited endothelial reactive oxygen species production [22]. Clinically, vitamin D replacement resulted in significant improvement in endothelial function, measured by flow mediated dilatation, after vitamin D_2_ replacement were observed in patients with Type 2 diabetes [23] and in healthy overweight African American adults [24]. In this study, we did not observe a change in ADMA concentrations in our vitamin D insufficient, but otherwise healthy volunteers, despite significant improvement in vitamin D concentrations after supplementation. It is entirely possible that in this younger cohort with no preexisting co-morbidities, ADMA clearance mechanisms via dimethylarginine diaminohydrolase (DDAH) enzyme is not oxidatively modified, and thus, restoration of vitamin D levels did not alter ADMA kinetics. Furthermore, while vitamin D supplementation resulted in marked decrease of TSP-1 levels, the correlation of the change in 25(OH)D levels and change in TSP-1 was not statistically significant, implying a possible complex biochemical interaction.

TSP-1 is a large matricellular glycoprotein, first identified as a protein released from α-granules of thrombin-stimulated platelets upon tissue injury [25]. TSP-1 regulates multiple cellular events involved in tissue repair including hemostasis, cell adhesion, migration, and proliferation (reviewed by [26]). It has been shown that TSP-1 inhibits NO-stimulated activation of sGC in endothelial cells, vascular smooth muscle cells, platelets, and T cells (reviewed by [[27]), with subsequent inactivation of cGMP-dependent protein kinases [28]. In addition to inhibiting NO/sGC signalling, TSP-1 can also inhibit NO production via disruption of the vascular endothelial growth factor pathway[29]. Furthermore, TSP-1 knockout vessels display greater endothelial-dependent relaxation compared to wild-type mice, and intravenous infusion of TSP-1 into rodents have been shown to raise blood pressure acutely [30]. However, we did not observe a relationship between change in TSP-1 and change in blood pressures in this cohort with vitamin D supplementation. While it remains possible that TSP-1 has effects on blood pressure, effects of vitamin D supplementation on blood pressure in this cohort may be predominantly via a different mechanism.

Elevated plasma concentrations of TSP-1 has been found in patients with coronary artery disease, diabetes mellitus [31], peripheral artery disease [32] and stroke [33]. Thus, elevated TSP-1 concentrations may be a biomarker of cardiovascular disease states. Currently, there are limited data that describe the association between TSP-1 with vitamin D. In human aortic smooth muscle cells, addition of vitamin D analogs calcitriol and paricalcitol led to marked down-regulation of PAI-1, TSP-1 mRNA and protein expression [34, 35]. In our study, we found that vitamin D supplementation significantly lowers TSP-1, but not PAI-1 concentrations. It is therefore, entirely possible that the effects of vitamin D in suppressing TSP-1 could have dual beneficial effects: 1) improving vascular NO signaling, and 2) prevention of TSP-1 activation of TGF-β1-mediated pro-inflammatory/pro-fibrotic signaling. 1,25-dihydroxyvitamin D, has been shown to completely abolish TGF-β1 -induced increase in TSP-1 in rat renal interstitial cells, with subsequent protection against renal interstitial myofibroblast activation [36]. Given these findings, we also sought to investigate biomarkers of TGF- β family members, activin A, and its antagonist FSTL3. It has been suggested that there is significant interplay between vitamin D and activin A [37, 38]; vitamin D response elements have been identified within the promoter region of the human Activin A gene [39]. However, in this study, 12 weeks of vitamin D supplementation did not change activin A or FSTL3 concentrations. We did observe a significant correlation between activin A and systolic blood pressure (R = 0.4, p = 0.04) after restoration of vitamin D status. Our results are consistent with previous studies that showed elevated activin A in patients with pulmonary hypertension [40] or preeclampsia [41], suggesting a potential role of activin A in regulating blood pressure.

This is a non-randomized, open label, pilot study not designed to measure cardiovascular outcomes, but aimed towards investigating relationship between vitamin D concentrations/effects and TSP-1 concentrations. The main limitation of this study remains in the ability to demonstrate cause-and-effect relationships conclusively in humans: at present such investigations can only be performed in cell culture or in small animal models. Nevertheless, the observed vitamin D effect on plasma TSP-1 levels was marked, robust and likely to be physiologically relevant. Furthermore, the blood pressure measurements were not blinded; however, given that the lowering of SBP correlated significantly with the increase in 25(OH)D levels after vitamin D supplementation, indicated that this is a robust relationship.

The role of vitamin D in blood pressure has been controversial. While vitamin D is an inhibitor of the renin-angiotension-aldosterone system [42], and low 25-hydroxyvitamin D levels have been associated with high BP in cross sectional studies [43, 44]; intervention studies have produced conflicting evidence on the BP-lowering effect of vitamin D. A recent comprehensive meta-analysis review found no evidence of BP reduction by supplementation with vitamin D or vitamin D analogues [45]. It is noteworthy that a majority of these studies were in patients with multiple co-existing conditions and background treatment, including preexisting antihypertensives and other cardiovascular medications [45], which could potentially mask the effects of vitamin D supplementation. It is entirely possible that the effect of vitamin D on blood pressure observed in our study is due to the fact that our patients are younger, normotensive, without preexisting cardiovascular or musculoskeletal comorbidities; and thus eliminating confounding factors that are present in other studies. While the effects of blood pressure lowering in our study appear robust, it is notable that our study was not placebo controlled and was not specifically designed to measure BP outcomes.

This study is the first demonstration in humans that any mainstream therapy can reduce TSP-1 levels *in vivo* and engender a reduction in systemic blood pressure. This is of particular interest given the apparent role of TSP-1 as a marker and mediator of vascular dysfunction [46]. We now describe that a simple, safe, cheap and commonly used therapy as vitamin D supplementation can reduce TSP-1 levels and potentially have beneficial effects on cardiovascular health. Given the widespread prevalence of vitamin D deficiency and its association with adverse cardiovascular events/disease states, this study provides a basis for further mechanistic basic science and clinical trial investigations into the regulation of TSP-1 by vitamin D and its potential therapeutic implications.

## Supporting information

S1 File Details of data collected. (XLS)
